# Evaluating the Practical Impact of Fast Microbiology on the Treatment of Bloodstream Infections: Real-World Evidence from a High-Complexity Infectious Disease Center

**DOI:** 10.3390/antibiotics15050457

**Published:** 2026-04-30

**Authors:** Maria Grazia Bocci, Stefania Cicalini, Giulia Capecchi, Sara Leone, Emanuela Caraffa, Giulia Valeria Stazi, Barbara Massa, Silvia D’Arezzo, Marina Selleri, Carla Fontana

**Affiliations:** 1 Intensive Care Unit, Clinical and Research Department, National Institute for Infectious Diseases “Lazzaro Spallanzani”—IRCCS, 00149 Rome, Italy; mariagrazia.bocci@inmi.it (M.G.B.); giulia.capecchi@inmi.it (G.C.); giuliavaleria.stazi@inmi.it (G.V.S.); 2 Systemic and Immune Depression-Associated Infections Unit, Clinical and Research Department, National Institute for Infectious Diseases “Lazzaro Spallanzani”—IRCCS, 00149 Rome, Italy; emanuela.caraffa@inmi.it (E.C.); barbara.massa@inmi.it (B.M.); 3 Clinical Epidemiology Unit, Department of Epidemiology, Preclinical Research and Advanced Diagnostic, National Institute for Infectious Diseases “Lazzaro Spallanzani”—IRCCS, 00149 Rome, Italy; sara.leone@inmi.it; 4 Laboratory of Microbiology and Biobank, National Institute for Infectious Diseases “Lazzaro Spallanzani”—IRCCS, 00149 Rome, Italy; silvia.darezzo@inmi.it (S.D.); marina.selleri@inmi.it (M.S.); carla.fontana@inmi.it (C.F.)

**Keywords:** bloodstream infections, rapid diagnostic tests, FAST microbiology, targeted therapy, diagnostic timeliness

## Abstract

Background/Objectives: Bloodstream infections (BSIs) are a major cause of morbidity and mortality, particularly when delays in pathogen identification hinder timely and targeted antimicrobial therapy. Rapid diagnostic tests (RDTs) accelerate microbiological identification, yet their clinical impact remains heterogeneous across different healthcare settings. This study aimed to evaluate the real-world effect of implementing FAST microbiology, a diagnostic workflow that integrates RDTs into conventional blood culture processing, on diagnostic timeliness, antimicrobial decision-making, and patient management in a hospital specializing in complex infectious diseases. Methods: We conducted a quasi-experimental study comparing non-FAST and FAST workflows over a 24-month period, including 166 adult patients with sepsis admitted to ICU and non-ICU units, accounting for 231 BSIs. Microbiological outcomes, treatment dynamics, time to targeted therapy, and key clinical endpoints were compared between non-FAST and FAST groups. Results: FAST microbiology significantly reduced the time to initiation of targeted therapy across clinical settings. No statistically significant differences were observed in hospital length of stay, overall mortality, or 28-day mortality between the two groups. Baseline clinical severity, age, and comorbidity burden remained the main determinants of clinical outcomes. Conclusions: These real-world findings support the integration of rapid diagnostics into existing antimicrobial stewardship frameworks by improving diagnostic timeliness and supporting earlier microbiologically guided therapeutic decisions. However, the results also highlight that accelerating diagnostics alone may not be sufficient to improve survival in critically ill patients with complex infectious diseases, where outcomes are predominantly driven by patient- and disease-related factors.

## 1. Introduction

Bloodstream infections (BSIs) pose a significant public health challenge, frequently leading to severe conditions such as sepsis and septic shock if not promptly identified and treated. Their rapid progression and association with high morbidity, mortality, and healthcare costs make BSIs a critical issue [1]. Blood culture (BC) remains the gold-standard, first-line diagnostic tool for BSIs, although the time required to obtain definitive results may delay the initiation of targeted antimicrobial therapy. Applying accelerated diagnostic workflows directly to positive blood cultures can improve diagnostic timeliness and support earlier treatment optimization [2,3]. In a clinical context, it is crucial to focus on the targeted use of antibiotics, alongside measures to prevent the spread of multidrug-resistant pathogens [4]. Antimicrobial resistance (AMR) is a growing concern, posing a serious threat to public health [5]. This concern is echoed in recent global governance analyses, such as the 2025 Lancet Infectious Diseases report, which found that countries with stronger implementation tools, particularly with regard to infection prevention and control, surveillance, and antimicrobial stewardship, experience significantly lower burdens of AMR-related mortality and disability [6].

Regular, day-to-day collaboration between microbiologists and clinicians in antimicrobial management is of paramount importance, particularly in critical care settings [7,8]. Timely discontinuation of antibiotics may be supported by diagnostic tests designed to identify pathogens from the initial positive blood culture. Detection may be further delayed by prior antibiotic exposure or the presence of fastidious organisms. Moreover, in some patients, symptom resolution alone is insufficient to justify antibiotic discontinuation [9]. Combining antimicrobial stewardship programs (AMS) with diagnostic stewardship (DS) is crucial [10]. DS involves selecting the appropriate diagnostic test for the right patient at the right time. This approach can significantly influence therapeutic choices, thereby supporting a more targeted AMS strategy [11]. Timely and accurate diagnosis is essential for identifying the causative pathogen and determining its susceptibility to specific antimicrobial agents. Earlier pathogen identification enables clinicians to initiate targeted antimicrobial therapy more rapidly [12].

Conversely, early targeted treatment can prevent infection progression and improve patient outcomes by promoting faster recovery, reducing complications, and ultimately lowering mortality rates [13]. It is important to recognize that outcomes in sepsis and BSIs are shaped by multiple host-, pathogen-, and context-related factors, including baseline clinical severity, comorbidity burden, immunological status, and microbiological complexity. These factors may inherently limit the extent to which diagnostic acceleration alone can modify survival. Although the literature supporting fast microbiology is extensive, and despite strong recommendations, the overall level of evidence reflected in guidelines remains moderate. This is largely attributable to limitations in most available randomized controlled trials (RCTs), including single-center designs, small sample sizes, short follow-up periods, and the use of composite outcomes. Observational studies also have several limitations and are more susceptible to bias and confounding; therefore, they cannot be used to demonstrate causality [14]. According to some studies, the implementation of fast microbiology may not be effective. Rapid diagnostic tests (RDTs) enable the prompt identification of bloodstream pathogens, providing clinicians with microbiological data in a timely manner. However, despite the potential benefits of this approach, current evidence indicates that RDTs alone do not translate into a clear reduction in patient mortality [15,16]. Hence, recommendations should be continuously updated to reflect emerging evidence from both clinical studies and real-world experience [14,16,17].

Evaluating the impact of rapid microbiology on BSIs within a hospital setting specializing in infectious disease care is particularly compelling. In such an environment, the history of complex infections and previous treatments is likely to have a significant impact on the effectiveness of rapid diagnostic tools.

At our institute, we adopt a broader approach to “FAST microbiology,” defined as an accelerated diagnostic workflow integrating RDTs with structured laboratory–clinician communication and established antimicrobial stewardship processes. This expanded FAST concept is particularly relevant in high-complexity infectious disease settings, where patients often present with severe infections, a substantial burden of comorbidities, and challenging microbiological profiles that may critically affect the translation of rapid diagnostics into clinical benefit. Therefore, this study aimed to assess the impact of FAST microbiology implementation on diagnostic timeliness, antimicrobial decision-making, and overall patient management, including its potential to improve therapeutic appropriateness and optimize clinical trajectories in a real-world, high-complexity environment.

## 2. Results

### 2.1. Overall Population

A total of 166 patients were included in the study, evenly distributed between the non-FAST (*n* = 84) and FAST (*n* = 82) phases (Table 1). During the FAST period, rapid diagnostic testing was not systematically applied to all eligible episodes: out of 335 blood cultures processed in this phase, 109 (32.5%) underwent FAST analysis, while the remaining 226 were managed through the conventional workflow. Baseline demographic and clinical characteristics did not differ meaningfully between patients who received FAST testing and those who did not. The cohort was predominantly male (57.2%), with no significant sex differences between the two groups. Patients in the non-FAST period were significantly older than those in the FAST period [median 74.0 years, interquartile range (IQR) 64.5–81.0 vs. 66.5 years, IQR 55.0–78.0; *p* = 0.014]. Most admissions originated from the ICU (73.5%), with similar proportions across both the non- and FAST periods. Comorbidity burden, as measured by the Charlson Comorbidity Index, was slightly higher in the non-FAST group than in the FAST group [3.5 (IQR 2.0–5.0) vs. 3.0 (IQR 1.0–4.0); *p* = 0.032]. The prevalence of individual comorbidities was generally comparable, except for hypertension (*p* = 0.005) and immunosuppression (*p* = 0.017), which were both more prevalent in the FAST patients (Table S1). Importantly, during the FAST implementation period, rapid diagnostic testing was not systematically applied to all eligible bloodstream infection episodes. Out of 335 blood cultures processed in this phase, 109 (32.5%) underwent FAST testing, while the remaining 226 were managed through the conventional workflow. No clinically meaningful baseline differences were observed between FAST-tested and non-tested patients, supporting the validity of subsequent comparative analyses.

The implementation of RDTs did not significantly affect clinical outcomes. Overall mortality was 39.2% in the entire population, with no statistically significant differences between groups (33.3% vs. 45.1% in the non- and FAST groups, respectively, *p* = 0.12). Similarly, 28-day mortality showed no significant variation (15.5% vs. 26.8%, respectively; *p* = 0.073). The length of hospital stay was also comparable among the non- and FAST groups, with median values of 40 days (IQR 20.5–55.0), and 29.5 days (IQR 13.0–56.0), respectively (*p* = 0.20).

At admission, the prevalence of COVID-19 diagnosis differed significantly between patients’ cohorts. Overall, 59.0% of patients enrolled in the study had a diagnosis of COVID-19 upon admission. However, this proportion was significantly lower in the FAST group (46.3%) than in the non-FAST group (71.4%) (*p* = 0.001), reflecting the differences in the epidemiological phases during the study period. Regarding BSI characteristics, most episodes were classified as isolated BSIs (69.9%). The distribution of BSIs was similar between non- and FAST groups (isolated BSIs: non-FAST vs. FAST 70.2% vs. 69.5%; *p* = 0.919).

### 2.2. Effect of FAST Microbiology on the Time to Initiation of Targeted Therapy

The analysis compared non-FAST and FAST periods, with the primary outcome being the time to initiation of targeted therapy. Because FAST testing was not systematically applied to all eligible bloodstream infection episodes during the implementation period, comparisons between the non-FAST and FAST groups reflect only those cases in which the accelerated diagnostic workflow was actually performed. This clarification ensures appropriate interpretation of the analyses that follow, including the evaluation of time to targeted therapy.

In the overall cohort of patients, the median time to initiation of therapy was significantly reduced in the FAST group *(p* < 0.001). In particular, the non-FAST group showed a median time of 3.0 days (IQR 3.0–4.0), whereas the FAST group achieved a median of 1.0 day (IQR 1.0–2.0; *p* < 0.001). When analyzed within individual clinical settings, a consistent benefit was observed across units. In the ICU population, the time to targeted therapy decreased from 3.0 days (IQR 3.0–4.0) in the non-FAST group to 1.0 day (IQR 1.0–2.0) in the FAST group (*p* < 0.001), with a subset of patients receiving treatment in less than 24 h. Similarly, in the Systemic and Immune Depression–Associated Infections Unit (non-ICU population, *n* = 56), the median time was shortened from 3.0 days (IQR 3.0–3.5) to 1.5 days (IQR 1.0–2.0; *p* < 0.001). The univariable Cox regression model showed a more than sevenfold higher likelihood of starting targeted therapy earlier with FAST implementation (HR 7.88, 95% CI 4.73–13.11; *p* < 0.001). After controlling for the a priori identified confounders of sex, ward, severity of admission SOFA score, age-adjusted Charlson Comorbidity Index, COVID-19 diagnosis at admission, BSI episodes and resistant pathogens, the effect of FAST implementation was even larger (HR 8.42, 95% CI 5.39–13.23; *p* < 0.001) (Table 2 and Table S2).

These findings demonstrate that rapid microbiology consistently and significantly reduced the time to initiation of targeted therapy across the entire cohort, with a more pronounced effect among ICU patients, a subgroup in which timely treatment was achieved in a meaningful proportion of cases.

**Figure 1:**
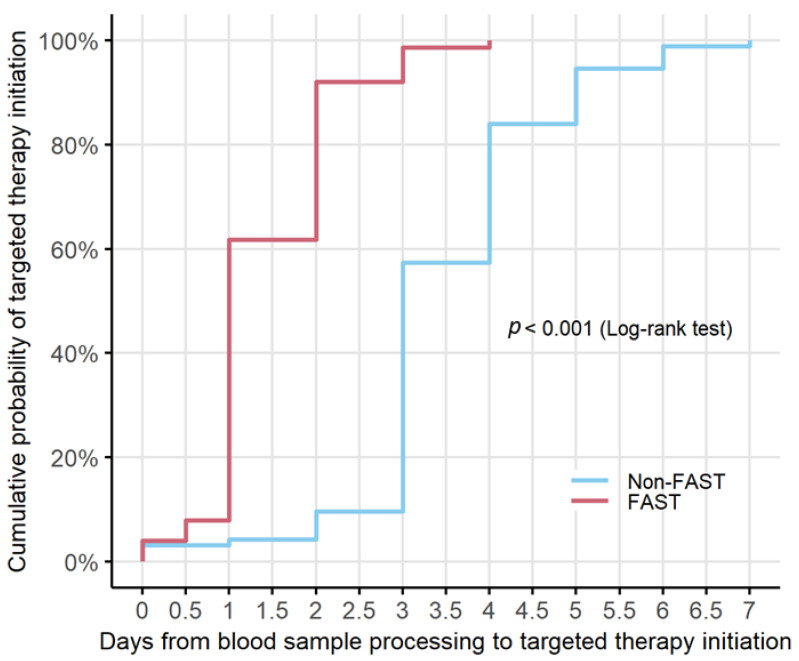
Kaplan–Meier curve showing the cumulative probability of targeted therapy initiation in the non-FAST (blue line) and FAST (red line) periods. Differences between groups were assessed using the Log-rank test (*p* < 0.001).

These results were further confirmed by the Kaplan–Meier analysis, which demonstrated a significantly shorter time to targeted antimicrobial therapy in the FAST cohort compared with the non-FAST cohort (Figure 1). Patients in the FAST period initiated targeted therapy earlier and with a higher cumulative probability over time. The difference between groups was statistically significant (log-rank test, *p* < 0.001). Notably, a substantial proportion of patients in the FAST cohort initiated appropriate antibiotic therapy within the first 24–48 h, whereas treatment initiation in the non-FAST cohort occurred later and progressed more gradually.

### 2.3. Epidemiology of Isolated Microorganisms in the Study Period

A total of 231 BSIs were recorded during the study period, yielding 255 causative microorganisms (109 Gram-positive bacteria, 121 Gram-negative bacteria, and 25 fungi). In case of polymicrobial infection, each isolated pathogen was analyzed and reported individually.

A comparison of microorganisms isolated from BSIs excluding fungi, between the non-FAST and FAST periods, revealed differences in the distribution of bacterial isolates across time (Table 3). Because FAST microbiology represents a diagnostic and communication-based strategy and does not influence the underlying epidemiology of bloodstream pathogens, these differences should be interpreted as purely descriptive findings reflecting temporal variability and case mix rather than an effect attributable to FAST implementation.

Specifically, among Gram negative isolates, the proportion of resistant strains, defined as carbapenem-resistant (CARBA-R) isolates or those harbouring extended spectrum β-lactamases (ESBL), was 32.8% (19/58) in the non-FAST period and 46.0% (29/63) in the FAST period, and this difference did not reach statistical significance (*p* = 0.136). Focusing on the main groups, the proportion of CARBA-R Enterobacterales was 26.7% (8/30) in the non-FAST period and 41.0% (16/39) in the FAST period, also without reaching statistical significance (*p* = 0.324). These proportions reflect resistance patterns observed over time in this clinically critical population. By contrast, the proportion of CARBA-R *Pseudomonas aeruginosa* strains was 40.0% (6/15) in the non-FAST period and 18.2% (2/11) in the FAST period (*p* = 0.395), consistent with known geographical and temporal variability in the spread of resistant strains and related clinical outcomes [18,19,20]. Regarding Gram-positive bacteria, the proportion of methicillin-resistant (MetR) *S. aureus* was 76.9% (10/13) in the non-FAST period and 50.0% (6/12) in the FAST period (*p* = 0.006), while the proportion of vancomycin-resistant enterococci (VRE) was 18.2% (10/55) and 9.2% (5/54) of total Gram-positive isolates in the non-FAST and FAST periods, respectively (*p* = 0.283). Overall, these findings describe resistance patterns observed across the two study periods and should be interpreted descriptively, as FAST microbiology does not influence the occurrence or epidemiology of antimicrobial resistance.

### 2.4. Impact of FAST Microbiology on Antibiotic Therapy Management

Patients were categorized into three groups according to the subsequent therapeutic decisions following the availability of microbiological results: (i) continuation of treatment, including patients who were receiving an empirical antibiotic regimen that remained unchanged after the microbiology results were received; (ii) change of treatment, including patients who were receiving empirical treatment before diagnosis and whose regimen was modified according to the microbiological results; and (iii) introduction of treatment, referring to patients who were not receiving any empirical antibiotics and who started a targeted regimen after the microbiological results were received. 

A total of 231 BSIs were included, of which 53% occurred in the non-FAST period and 47% in the FAST period (Table 4).

BSI episodes between the ICU and non-ICU settings were similar across phases, with no significant differences observed (*p* = 0.456). Overall, most of the patients in the cohort originated from the ICU. In contrast, therapeutic decisions differed significantly between the two periods (*p* = 0.001). The proportion of patients for whom treatment was continued without modification, and from whom targeted therapy was initiated, was significantly higher in the FAST phase, with no statistically significant difference between groups in the pairwise comparisons (*p* = 0.394). Conversely, the proportion of patients requiring a change of treatment decreased markedly after the implementation of FAST microbiology (60.7% vs. 37.6%), compared with those who continued with the same treatment (*p* = 0.042) and those who initiated a new treatment (*p* = 0.001). These findings indicate that FAST microbiology contributed to greater diagnostic and therapeutic confidence, enabling clinicians to maintain or target therapy more effectively and reducing reliance on treatment modifications after initial management.

### 2.5. Effect of FAST Microbiology Implementation on Overall Mortality

A univariable logistic regression model showed no statistically significant evidence of an increased odds ratio for overall mortality during FAST implementation compared with non-FAST implementation (OR 1.64, 95% CI 0.88–3.10; *p* = 0.121). After adjusting for potential confounding factors, including sex, ward, SOFA score at admission, age-adjusted Charlson Comorbidity Index, diagnosis of SARS-CoV-2 at admission, bloodstream infection episodes and resistant pathogens, the results were similar (OR 1.94, 95% CI 0.93–4.13; *p* = 0.079) (Table 5 and Table S3).

## 3. Discussion

Sepsis remains one of the leading causes of morbidity and mortality worldwide, and the timely initiation of targeted antimicrobial therapy is a cornerstone of effective management [21]. In recent years, RDTs have been introduced to accelerate pathogen identification, shorten time to targeted treatment, and potentially improve patient outcomes. Although the benefits of these technologies are well established, it is uncertain whether they improve patient survival, especially in complex, high-risk settings such as ICUs [22,23].

Implementing FAST microbiology in our high-complexity infectious diseases center resulted in a marked improvement in diagnostic and therapeutic process indicators. Most notably, the time to targeted antimicrobial therapy was substantially reduced. These findings are consistent with evidence-based laboratory medicine guidelines and multiple systematic reviews demonstrating that rapid diagnostic technologies reliably accelerate pathogen identification and enhance the timeliness and appropriateness of antimicrobial decision-making [23,24,25]. It is important to note that FAST microbiology was not introduced into an unstructured or suboptimal system, rather, its implementation occurred within a mature and well-coordinated antimicrobial stewardship (AMS) framework characterized by regular interdisciplinary review of microbiological data and established therapeutic pathways. Accordingly, the clinical value of FAST microbiology should be interpreted primarily in terms of diagnostic timeliness and support for antimicrobial decision making rather than differences in pathogen distribution, with particular relevance for pathogens and resistance phenotypes for which early identification has the greatest therapeutic impact.

Mortality analysis was primarily driven by disease severity at admission and by the complexity of BSIs, as reflected by the independent associations of higher SOFA score at admission and repeated or polymicrobial BSI episodes. These findings are consistent with current understanding of sepsis pathophysiology, where both the initial burden of organ dysfunction and ongoing infectious complications play a central role in determining outcomes. The apparent protective effect associated with admission to the non-ICU ward should be interpreted with caution, as it may reflect differences in patient severity than a true causal benefit. Similarly, the lack of a statistically significant effect of FAST microbiology on mortality, despite a trend toward higher odds, reflects clinical selection bias, as patients with greater clinical severity were more likely to undergo rapid diagnostic testing. The lack of an independent association with antimicrobial resistant pathogens causative of BSIs suggests that patient-related factors and the complexity of the infection may play a more important role than microbiological characteristics alone in determining prognosis in this setting. Overall, these results highlight the importance of considering disease severity and infection complexity in risk stratification and also emphasize the need to carefully interpret observational data, especially when assessing the impact of diagnostic interventions.

In such a highly optimized environment, the incremental impact of further diagnostic acceleration on hard clinical endpoints, such as mortality, is inherently limited, given that baseline stewardship performance is already high. However, the higher mortality observed after FAST implementation can be attributed to several non-significant yet clinically meaningful differences between the cohorts, including a higher prevalence of immunosuppression, subtle shifts in the epidemiological context, and a trend toward more complex microbiological profiles.

Indeed, multicenter studies have reported 30-day mortality rates of up to 36–38% for infections caused by CARBA-R Enterobacterales [18,20,26]. The presence of CARBA-R Enterobacterales poses a significant therapeutic challenge due to the severe limitation of available treatment options and its potential contribution to increased mortality rates in the post-intervention period. Several studies have demonstrated that infections caused by these pathogens are associated with poorer clinical outcomes and significantly higher mortality rates than those caused by susceptible strains [18,20].

Moreover, the absence of statistical significance does not imply the absence of a true underlying effect, particularly in studies with limited sample size and statistical power, where moderate or heterogeneous effects may remain undetected. Within this framework, the increased mortality observed in the FAST period should be interpreted as a reflection of the complexity surrounding the patients and pathogens involved, rather than being attributed to the diagnostic intervention itself.

This interpretation aligns with the broader literature. Several evaluations indicate that improvements in diagnostic turnaround time do not necessarily translate into measurable survival benefits in real-world clinical settings. Systematic reviews have shown that while rapid molecular assays consistently shorten time to effective therapy, their impact on mortality is heterogeneous and context-dependent [27]. Likewise, meta-analytic evidence demonstrates that mortality benefits associated with rapid diagnostics are not uniformly observed across diverse patient populations, especially in heterogeneous cohorts with severe BSIs [28]. These converging findings highlight a widely recognized principle: while rapid diagnostics can improve process metrics, they are not sufficient on their own to alter survival outcomes in critically ill or clinically complex patients [27,28]. 

Overall, our results reinforce that FAST microbiology provides significant value within an already high-performing AMS environment by enhancing diagnostic efficiency, improving the timeliness of therapeutic decisions, and strengthening clinical confidence. However, the multifactorial nature of mortality in severe BSIs driven by host characteristics, comorbidity burden, pathogen virulence, and source control likely outweighs the incremental effects of diagnostic acceleration alone. The absence of a detectable mortality benefit should therefore be interpreted not as a limitation of FAST itself, but as a reflection of the complex and multidimensional determinants of survival in critically ill patients.

This study has some limitations. Firstly, the study period included both the late-pandemic and post-pandemic phases of the SARS-CoV-2 pandemic. This may have introduced epidemiological variability that could have influenced patient demographics, baseline risk factors, and case mix. Although COVID-19 was included in the multivariable analysis and was not independently associated with mortality, its impact cannot be fully excluded. COVID-19 may have affected disease severity, clinical management, and diagnostic strategies in ways not entirely captured by the variables included in our model. Therefore, residual confounding related to temporal changes in patient characteristics and healthcare context remains possible and should be considered when interpreting these findings.

Secondly, while the sample is representative of a high-complexity center, the relatively small sample size may have limited the statistical power to detect differences in outcomes such as mortality. Consequently, non-statistically significant findings should be interpreted with caution, as clinically relevant effects cannot be ruled out. Future larger, adequately powered studies are warranted.

Thirdly, the monocentric design may restrict generalizability, as our institution benefits from a highly structured and mature AMS program; the incremental impact of FAST microbiology might differ in settings with lower baseline stewardship performance or different organizational models.

Fourthly, although FAST was available throughout the post-implementation period, clinicians may have applied it preferentially to more complex or unstable patients, introducing a degree of indication bias that could attenuate the measurable effects on survival.

Finally, an additional limitation of this study is the absence of detailed data on antibiotic consumption and de-escalation practices, as these variables were not systematically collected. This may limit a comprehensive assessment of the impact of FAST implementation on antimicrobial prescribing patterns.

Overall, residual confounding cannot be excluded. Several clinically relevant differences between the non-FAST and FAST cohorts, even when not statistically significant, may have influenced outcomes, including variations in immunosuppression, subtle epidemiological changes, and a higher microbiological complexity in the post-intervention period. These limitations highlight the need for adequately powered, prospective, multicenter studies to more definitively assess the clinical impact of FAST microbiology on patient-centered outcomes.

## 4. Materials and Methods

### 4.1. Setting

The National Institute for Infectious Diseases ‘Lazzaro Spallanzani’ is a healthcare and research institute, with approximately 200 beds, including 17 intensive care beds, and extensive experience in managing patients with complex infectious diseases.

### 4.2. Study Site and Design

The study was conducted at the Systemic and Immune Depression-Associated Unit (non-ICU) and the Intensive Care Unit (ICU) at the National Institute for Infectious Diseases L. Spallanzani. It included adult patients (≥18 years) admitted with a diagnosis of BSI. The study used a quasi-experimental pre-post design, comparing data collected before and after the implementation of RDTs. It was conducted from March 2022 to February 2024 and divided into two phases: March 2022–February 2023 (non-FAST) and March 2023–February 2024 (FAST). For this study, FAST microbiology was operationally defined as an accelerated diagnostic workflow in which RDTs are systematically integrated into conventional blood culture processing and embedded within structured laboratory–clinician communication pathways and established antimicrobial stewardship practices, aimed at enhancing diagnostic efficiency and optimizing patient management.

During the non-FAST phase, microbiological evaluation relied exclusively on conventional culture-based blood testing. In the FAST phase, in addition to standard culture methods, a fast-microbiology approach was made available. The decision to apply FAST methods was based on clinical judgement and availability, reflecting real-world practice.

### 4.3. Microbiology Pathways

Blood samples were obtained from adult patients diagnosed with sepsis and were inoculated in both aerobic and anaerobic BC bottles (BACTEC Plus/F Aerobic) and into Bactec Plus Anaerobic/f bottles and incubated in the BD BACTEC FX system (Becton Dickinson, Sparks, MD, USA) (BACTEC Plus Aerobic/F and BACTEC Plus Anaerobic/F; Becton Dickinson, Sparks, MD, USA) and incubated in the BD BACTEC FX system (Becton Dickinson, Sparks, MD, USA).

For clarity, the two study phases are referred to as non-FAST (conventional workflow) and FAST (conventional workflow plus rapid diagnostic tests), corresponding to the pre implementation and post implementation periods, respectively.

FAST testing was implemented according to clinical judgement and operational availability. Consequently, only 109 of the 335 eligible blood cultures in the FAST period underwent the FAST workflow. This variability reflects real-world practice and has been explicitly acknowledged in the analysis.

Non-FAST workflow: The blood culture bottles were processed by microbiologists. Bottles flagged as positive were removed from the incubators and processed. Gram staining was performed for positive blood cultures, and the preliminary results would be reported electronically to the medical recording system. At the same time, an aliquot of broth culture from positive BCs was plated onto targeted solid media: blood agar, chocolate agar, MacConkey agar, Chapman agar, and Sabouraud-dextrose agar (Thermofisher, Basingstoke, UK) using Walk Away Specimen Processor (WASP) (Copan Brescia, Brescia, Italy) (WASP; Copan Italia, Brescia, Italy). Plates were incubated at 37 °C, 5% CO_2_ for 24–48 h. Microbial isolates were identified using a MALDI-TOF MS Syrius (Bruker Daltonics, Bremen, Germany). While the antimicrobial susceptibility testing of the isolates was performed using a semi-automated Phoenix system (Becton Dickinson Diagnostics, San Diego, CA, USA), MICs for colistin, ceftazidime/avibactam, and cefiderocol were confirmed by the broth microdilution method using the ComASP^®^ system (Liofilchem, Roseto degli Abruzzi, Italy), while for meropenem/vaborbactam, the MTS™ MIC Test Strip (Liofilchem, Roseto degli Abruzzi, Italy) while MICs for meropenem/vaborbactam were determined using MTS™ MIC Test Strips (Liofilchem, Roseto degli Abruzzi, Italy). Results were interpreted according to the European Committee on Antimicrobial Susceptibility Testing guidelines (EUCAST, 2020–2023). The detection and identification of KPC, VIM, IMP, NDM, and OXA-48-like carbapenemases were achieved using an immunochromatographic assay (NG-Test CARBA 5, Biotech, Guipry, France). The laboratory’s final report was conveyed to the clinician via the laboratory information system (LIS, software details not specified). In the event of detection of significant pathogens, such as Enterobacterales carbapenem-resistant, *P. aeruginosa* multi-drug-resistant (MDR), and methicillin-resistant *S. aureus* (MRSA), VRE, and *Acinetobacter baumannii* MDR, an alert is sent to the clinicians as well as to the infection prevention and control team and the health director.

FAST workflow: During the FAST period, the microbiology pathway was modified by introducing rapid identification techniques alongside the conventional culture-based workflow. Specifically, pathogen identification was performed using the MBT Sepsityper^®^ IVD Kit (Bruker Daltonics, Bremen, Germany) and/or the BioFire^®^ FilmArray^®^ Blood Culture Identification (BCID) Panel (bioMérieux, Marcy-l’Étoile, France) following blood culture positivity. In this study, rapid identification within the FAST workflow was performed on positive blood culture broth using MALDI-TOF-based and/or multiplex molecular assays, and not directly on whole blood samples.

The result obtained with the MBT Sepsityper^®^, when available, was preferentially reported to the clinician via the laboratory information system (LIS; software details not specified) and communicated by telephone in the case of clinically relevant pathogens. During the FAST period, microbiological results and related clinical and therapeutic data were systematically reviewed during regular interdisciplinary meetings involving microbiologists and clinicians from the participating units, as part of the established antimicrobial stewardship framework.

### 4.4. Outcome Variables Definition

The primary outcome of the study was the time to initiation of targeted therapy. For this analysis, “targeted therapy” was operationally defined as any antimicrobial regimen that was started or modified based on microbiological findings, specifically organism identification and, when available, susceptibility data, obtained through the conventional or FAST workflow. Accordingly, the primary outcome measured the interval between (i) the arrival of the blood sample in the microbiology laboratory and its processing, and (ii) the communication to clinicians of blood culture positivity together with the corresponding identification result that enabled a microbiologically guided therapeutic decision.

Following receipt of microbiological results, each bloodstream infection (BSI) episode was assigned to one of three mutually exclusive therapeutic categories:

(i) Continuation of treatment, referring to patients who were already receiving an empirical antimicrobial regimen that remained appropriate based on microbiological findings and therefore required no modification;
(ii) Change of treatment, referring to patients whose empirical therapy was adjusted according to the organism identified and/or susceptibility profile;
(iii) Introduction of treatment, referring to patients who were not receiving any empirical antimicrobial therapy at the time of blood culture positivity and for whom treatment was started following microbiological results.

Because patients in the “continuation of treatment” category did not require the initiation of microbiologically targeted therapy, they were excluded from all analyses specifically designed to evaluate the impact of FAST microbiology on time to initiation of targeted therapy. This ensured that the primary outcome captured the true contribution of diagnostic acceleration to clinically meaningful therapeutic decisions.

Therapeutic categorization was performed independently and blinded to clinical outcomes to minimize misclassification bias. In addition to the primary outcome, secondary clinical endpoints—such as all-cause mortality and length of hospital stay—were explored to assess potential downstream effects of FAST microbiology on patient prognosis.

### 4.5. Analysis of Isolated Microorganisms

Microbiological data were extracted from the dataset. Each patient could have one or more BSI; for this study, each organism isolated from blood cultures was recorded as a single isolate event, such that individual patients could contribute to multiple events (including polymicrobial cultures). Isolated events were first summarized overall and then stratified into non- and FAST periods. All microorganisms were initially categorized as Gram-positive, Gram-negative, or fungi; fungal isolates were excluded from all subsequent analyses. Bacterial isolates were further classified as follows: among Gram-positive, *Staphylococcus aureus*, coagulase-negative staphylococci (CoNS), *Enterococcus faecium*, and *Enterococcus faecalis,* other *Enterococcus* species; among Gram-negative, non-fermenting Gram-negative bacilli (NFGNB) and Enterobacterales. Resistance phenotypes were derived from routine antimicrobial susceptibility testing. Among Gram-positive isolates, methicillin resistance was assessed for staphylococci, whereas vancomycin resistance was assessed for enterococci; for Gram-negative isolates, carbapenem resistance and ESBL production were recorded.

### 4.6. Statistical Analysis

Continuous variables were expressed as median with interquartile range (IQR) and were compared using the Wilcoxon rank sum test. Categorical variables were expressed as absolute frequency with percentage and were compared using the Chi-squared or Fisher’s exact test, as appropriate. For multiple comparisons, the Benjamini–Hochberg method was used for post hoc testing.

Cumulative probabilities of target therapy initiation were estimated using the Kaplan–Meier method and were compared between the non-FAST and FAST period using the log-rank test. Univariable and multivariable Cox regression models were performed to estimate the association between the non- and FAST period and the time to target therapy initiation. Both time-to-event analyses accounted for clustering on patient ID using robust standard errors. We also investigated the association between the non- and FAST period and the overall mortality by fitting univariable and multivariable logistic regression models. All multivariable models were adjusted for the following baseline covariates–identified a priori as potential confounders: sex, ward, severity of admission SOFA score, age-adjusted Charlson Comorbidity Index, COVID-19 diagnosis at admission, BSI episodes and resistant pathogens. A *p*-value of <0.05 was considered statistically significant. Data analysis was performed using R Statistical Software (version 4.2.1; R Foundation for Statistical Computing, Vienna, Austria).

## 5. Conclusions

In this real-world pre–post evaluation study, conducted in a high-complexity infectious diseases center, the implementation of FAST microbiology substantially improved key diagnostic and therapeutic processes, most notably by reducing the time to targeted therapy and supporting more timely antimicrobial decision-making. These findings reinforce the operational and clinical value of rapid microbiological diagnostics when integrated into a mature antimicrobial stewardship framework. However, despite these clear process improvements, no reduction in mortality was observed. This outcome should be interpreted within the broader context of severe BSIs, where survival is shaped predominantly by patient-related factors, comorbidity burden, pathogen virulence, and source control rather than by diagnostic acceleration alone. The observed mortality pattern reflects the intrinsic complexity of the population rather than any adverse effect of FAST itself.

Overall, our findings indicate that FAST microbiology enhances the quality and efficiency of care. However, its effect on major clinical outcomes appears limited in settings where antimicrobial stewardship programs and clinical practices are already highly optimized. Larger, adequately powered multicenter studies are therefore needed to better define the contribution of FAST diagnostic strategies to meaningful, patient-centred outcomes.

## Figures and Tables

**Table 1:** Demographic and clinical description of the patients included in the study.

Variable	Overall	Non-FAST	FAST	*p*-Value ^1^
	*n* = 166 (100%)	*n* = 84 (51%)	*n* = 82 (49%)	
Sex (*n*, %)				0.335
Male	95 (57.2%)	45 (53.6%)	50 (61.0%)	
Female	71 (42.8%)	39 (46.4%)	32 (39.0%)	
Age in years (Median, IQR)	71.0 [59.0, 79.0]	74.0 [64.5, 81.0]	66.5 [55.0, 78.0]	0.014
Age class (*n*, %)				0.366
25–49	18 (10.8%)	7 (8.3%)	11 (13.4%)	
50–75	90 (54.2%)	44 (52.4%)	46 (56.1%)	
+75	58 (34.9%)	33 (39.3%)	25 (30.5%)	
Ward (*n*, %)				0.796
ICU	122 (73.5%)	61 (72.6%)	61 (74.4%)	
Non-ICU	44 (26.5%)	23 (27.4%)	21 (25.6%)	
Admission SOFA score (Median, IQR)	6.0 [4.0, 9.0]	6.0 [5.0, 9.0]	6.0 [3.0, 9.0]	0.16
Severity of admission SOFA score (*n*, %)				0.218
≤5	71 (42.8%)	32 (38.1%)	39 (47.6%)	
≥6	95 (57.2%)	52 (61.9%)	43 (52.4%)	
Charlson Comorbidity Index (Median, IQR)	3.0 [2.0, 5.0]	3.5 [2.0, 5.0]	3.0 [1.0, 4.0]	0.032
Comorbidities (*n*, %)				0.718
No	7 (4.2%)	3 (3.6%)	4 (4.9%)	
Yes	159 (95.8%)	81 (96.4%)	78 (95.1%)	
Clinical outcome (*n*, %)				
Overall mortality	65 (39.2%)	28 (33.3%)	37 (45.1%)	0.12
28-day mortality	35 (21.1%)	13 (15.5%)	22 (26.8%)	0.073
Length of stay (Median, IQR)	34 [17.0,55.0]	40 [20.5, 55.0]	29.5 [13.0,56.0]	0.2
COVID-19 diagnosis at admission (*n*, %)				0.001
No	68 (41.0%)	24 (28.6%)	44 (53.7%)	
Yes	98 (59.0%)	60 (71.4%)	38 (46.3%)	
BSI episodes (*n*, %)				0.919
Isolated BSI	116 (69.9%)	59 (70.2%)	57 (69.5%)	
Repeated BSIs	50 (30.1%)	25 (29.8%)	25 (30.5%)	

^1^ Pearson’s Chi-squared test; Wilcoxon rank sum test; Fisher’s exact test.

**Table 2:** Cox regression analysis on the time to initiation of targeted therapy.

Characteristic	Univariable Cox Regression				Multivariable Cox Regression *		
	N	HR ^1^	95% CI ^1^	*p*-Value	HR ^1^	95% CI ^1^	*p*-Value
**Implementation period**	170						
**non-FAST**		—	—		—	—	
**FAST**		7.88	4.73, 13.11	<0.001	8.45	5.39, 13.23	<0.001

* Models adjusted for sex, ward, severity of admission SOFA score, age-adjusted Charlson Comorbidity Index, COVID-19 diagnosis at admission, BSI episodes and resistant pathogens; ^1^ HR = Hazard Ratio, CI = Confidence Interval.

**Table 3:** Isolated microorganisms and antibiotic resistance.

		Non-FAST			FAST			
Organism(s) Isolated		Total *n* (%)	MetR *^b^* *n* (%)	VRE *^c^* *n* (%)	Total *n* (%)	MetR *n* (%)	VRE *n* (%)	*p*-Value
	*Staphylococcus aureus*	13 (24)	10 (63)	-	12(22)	6 (67)	-	0.006 ^1^
	CoNS *^a^*	10 (18)	6 (37)	-	15 (28)	3 (33)	-	
	*Enterococcus faecalis*	21 (38)	-	6 (60)	11 (20)	-	3 (60)	
	*Enterococcus faecium*	4 (7)	-	2 (20)	10 (18)	-	2 (40)	
	Other *Enterococcus* spp.	2 (4)	-	2 (20)	0	-	-	
Other Gram-positive bacteria	-	5 (9)	-	-	6 (11)	-	-	
Total Gram-positive bacteria	-	55	16	10	54	9	5	0.283 ^2^
**Organism(s) Isolated**		**Total** ***n*** **(%)**	**CARBA-R *^e^*** ***n* ** **(%)**	**ESBL *^f^*** ***n* ** **(%)**	**Total** ***n*** **(%)**	**CARBA-R *n* (%)**	**ESBL** ***n*** **(%)**	***p*** **-Value**
NFGNB *^d^*	*Acinetobacter baumannii*	5 (9)	4 (21)	0	8 (13)	8 (31)	0	
	*Pseudomonas aeruginosa*	15 (26)	6 (32)	0	11 (17)	2 (8)	1 (25)	0.395 ^3^
	*Stenotrophomonas maltophilia*	5 (9)	1 (5) *^g^*	1 (50)	2 (3)	0	0	
	*Aeromonas hydrophila*	0	0	0	2 (3)	0	0	
Enterobacterales	*Citrobacter koseri*	1 (2)	0	0	0	0	0	0.324 ^4^
	*Enterobacter aerogenes*	1 (2)	0	0	0	0	0	
	*Enterobacter cloacae*	1 (2)	0	0	0	0	0	
	*Enterobacter hormaechei*	0	0	0	2 (3)	0	0	
	*Escherichia coli*	4 (7)	0	0	10 (16)	0	2 (50)	
	*Klebsiella aerogenes*	1 (2)	0	0	0	0	0	
	*Klebsiella oxytoca*	1 (2)	0	0	1 (2)	0	0	
	*Klebsiella pneumoniae*	16 (28)	8 (42)	1(50)	19 (30)	15 (58)	1 (25)	
	*Klebsiella variicola*	0	0	0	2 (3)	1 (4)	0	
	*Proteus mirabilis*	2 (3)	0	0	1 (2)	0	0	
	*Salmonella* spp.	0	0	0	2 (3)	0	0	
	*Serratia marcescens*	3 (5)	0	0	2 (3)	0	0	
Other Gram-negative bacteria	-	3 (5)	0	0	1 (2)	0	0	
Total Gram-negative bacteria	-	58	19	2	63	26	4	0.136 ^5^

*^a^* Coagulase-negative staphylococci; *^b^* Methicillin-resistant; *^c^* Vancomycin-resistant enterococci; *^d^* Non-fermenting Gram-negative bacteria; *^e^* Carbapenem-resistant; *^f^* Extended spectrum beta-lactamases; *^g^* Carbapenem susceptibility testing is not recommended for *S. maltophilia* due to intrinsic resistance (EUCAST); results, when available, should not be clinically interpreted. ^1^ Fisher’s Exact test compared MetR *S. aureus* between non- and FAST periods. ^2^ Pearson’s Chi-squared test compared VRE between non- and FAST periods. ^3^ Fisher’s Exact test compared CARBA-R *P. aeruginosa* between non- and FAST periods. ^4^ Pearson’s Chi-squared test compared CARBA-R Enterobacterales between non- and FAST periods. ^5^ Pearson’s Chi-squared test compared Gram-negative resistant isolates between non- and FAST periods.

**Table 4:** Effect of FAST microbiology techniques on antibiotic treatment optimization.

Variable (*n*, %)	Overall	Non-FAST	FAST	*p*-Value
	*n* = 231 (100%) ^1^	*n* = 122 (53%) ^1^	*n* = 109 (47%) ^1^	
Ward				0.456
ICU	175 (75.8%)	90 (73.8%)	85 (78.0%)	
Non-ICU	56 (24.2%)	32 (26.2%)	24 (22.0%)	
Therapeutic decision				0.001
Continuation of treatment	61 (26.4%)	28 (23.0%)	33 (30.3%)	
Change of treatment	115 (49.8%)	74 (60.7%) ^†,‡^	41 (37.6%)	
Introduction of treatment	55 (23.8%)	20 (16.4%)	35 (32.1%)	

^1^ Pearson’s Chi-squared test; ^†^ *p*-value < 0.05, comparison between change of treatment and continuation of treatment (adjusted with Benjamini–Hochberg method; ^‡^ *p*-value < 0.05, comparison between change of treatment and introduction of treatment (adjusted with Benjamini–Hochberg method).

**Table 5:** Logistic regression analysis on mortality.

Characteristic	Univariable Logistic Regression				Multivariable Logistic Regression *		
	N	OR ^1^	95% CI ^1^	*p*-Value	OR ^1^	95% CI ^1^	*p*-Value
**Implementation period**	166						
**non-FAST**		—	—		—	—	
**FAST**		1.64	0.88, 3.10	0.121	1.94	0.93, 4.13	0.079

* Models adjusted for sex, ward, severity of admission SOFA score, age-adjusted Charlson Comorbidity Index, COVID-19 diagnosis at admission, BSI episodes and resistant pathogens; ^1^ OR = Odds Ratio, CI = Confidence Interval.

## Data Availability

The data supporting the findings of this study are available upon reasonable request. Due to the sensitive and potentially identifiable nature of the clinical information derived from hospitalized patients, the dataset cannot be made publicly accessible. The corresponding author may provide anonymized data upon justified request, subject to approval by the National Institute for Infectious Diseases ‘Lazzaro Spallanzani’ IRCCS, and in compliance with applicable ethical and privacy regulations.

## Supplementary Materials

The following supporting information can be downloaded at: https://www.mdpi.com/article/10.3390/antibiotics15050457/s1, Table S1: Patient’s comorbidities; Table S2: Cox proportional hazards model for time to initiation of targeted therapy; Table S3: Logistic regression analysis on mortality.

## Abbreviations

The following abbreviations are used in this manuscript:

BSI	Bloodstream infection
RDTs	Rapid diagnostic tests
BC	Blood culture
AMR	Antimicrobial resistance
AMS	Antimicrobial stewardship
DS	Diagnostic stewardship
RCT	Randomized controlled trial
CARBA-R	Carbapenem resistance
ESBL	Extended spectrum beta-lactamases
VRE	Vancomycin-resistant enterococci
Met-R	Methicillin-resistant
LIS	Laboratory information system
MDR	Multi-drug-resistant
MRSA	Methicillin-resistant *S. aureus*
CoNS	Coagulase-negative staphylococci
NFGNB	Non-fermenting Gram-negative bacilli
